# Evaluation of iRoot BP Plus for Pulpotomy in Permanent Anterior Teeth With Complicated Crown Fractures in Pediatric Patients: A Prospective Cohort Study

**DOI:** 10.1155/ijod/1127489

**Published:** 2025-10-09

**Authors:** Jiajia Zheng, Xue Yang, Yuan Fu, Bichen Lin, Bingqing Shi, Xiao Shao, Quan Wen

**Affiliations:** ^1^First Clinical Division, Peking University School and Hospital of Stomatology and National Center for Stomatology and National Clinical Research Center for Oral Diseases and National Engineering Laboratory for Digital and Material Technology of Stomatology and Beijing Key Laboratory of Digital Stomatology and Research Center of Engineering and Technology for Computerized Dentistry Ministry of Health and NMPA Key Laboratory for Dental Materials, Beijing 100034, China; ^2^Department of Oral Emergency, Peking University School and Hospital of Stomatology and National Center for Stomatology and National Clinical Research Center for Oral Diseases and National Engineering Laboratory for Digital and Material Technology of Stomatology and Beijing Key Laboratory of Digital Stomatology and Research Center of Engineering and Technology for Computerized Dentistry Ministry of Health and NMPA Key Laboratory for Dental Materials, Beijing 100081, China

**Keywords:** bioactive ceramics, complicated crown fracture, incisor, pulpotomy

## Abstract

**Objective:**

This study aims to assess the effectiveness of pulpotomy in treating permanent anterior teeth with complicated crown fractures in children. Additionally, it seeks to analyze the long-term outcomes to inform clinical practices for dental trauma across a wider age spectrum.

**Methods:**

A total of 126 permanent incisors in 107 patients, aged 7–15 years, diagnosed with complicated crown fractures, were treated with pulpotomy using iRoot BP Plus as the pulp capping agent. The treated teeth underwent clinical assessments at 6–8 weeks, 3 months, 6 months, and 1 year post-treatment, followed by routine oral examinations. Outcomes were determined based on clinical and radiographic criteria assessed by calibrated examiners. Clinical examinations included assessment of root apex formation, mobility, crown color, pulp vitality test, and the presence of abscesses and fistulas. Radiographic examinations included evaluation of periodontal ligament continuity, periapical translucency, dentin bridge formation, and calcification of the pulp chamber and root canal.

**Results:**

With an average follow-up of 14.1 ± 12.6 months, success rates of 95.2% at 6 months and 90.9% at 12 months were observed. The estimated 2-year survival rate was 94.7% ± 2.7%. Success was independent of factors like gender, root maturation, pulpotomy type, pulp exposure time, and tooth mobility. Seven failures were noted, primarily due to periapical periodontitis. One case of pulp canal obliteration was observed.

**Conclusion:**

Pulpotomy using iRoot BP Plus shows a high success rate in children with nondisplaced complicated crown fractures in permanent teeth. The prognosis is unaffected by root apex formation or tooth mobility.

## 1. Introduction

A complicated crown fracture refers to a type of dental trauma where the enamel and dentin are fractured, resulting in pulp exposure [1]. In this condition, the pulp is directly exposed to the contaminated environment of the oral cavity. Epidemiological studies indicate that dental trauma affects 25%–33% of children and adolescents, with complicated crown fractures accounting for 2%–13% of these injuries [2, 3]. Permanent anterior teeth are particularly vulnerable due to their position and developmental stage, making this a significant clinical concern in pediatric dentistry.

In the past, root canal treatment was commonly used for mature permanent anterior teeth with pulp exposure to remove all vital pulp [4]. However, this approach reduces fracture resistance, impairs proprioception, and requires the removal of a substantial amount of tooth structure during access cavity preparation, which greatly affects the survival rate of the tooth after pulp therapy [5]. Moreover, losing vital pulp in young permanent anterior teeth halts root development, increasing susceptibility to brittleness and early tooth loss. This can lead to esthetic compromise, functional deficits, and psychological distress during critical developmental years.

The current philosophy emphasizes esthetics, function, and minimally invasive treatment as the three major aspects to consider in the treatment of traumatized anterior teeth [6]. Based on the minimally invasive concept, vital pulp therapy has become a new research focus [7]. Clinical consensus supports preserving vital pulp in mature permanent teeth after trauma when possible [8, 9].

Pulpotomy (partial or full) is indicated for complicated crown fractures, with the extent of pulp removal dictated by the pulp exposure size and infection level. While calcium hydroxide was historically used as a capping material in pulpotomy, its solubility and tendency to form porous dentin bridges limit long-term success [10, 11]. Bioactive materials like calcium silicate-based ceramics (e.g., iRoot BP Plus) promote denser dentin bridge formation and offer antibacterial properties, making them promising for pulpotomy [12]. While pulpotomy is endorsed by the International Association of Dental Traumatology (IADT) guidelines for managing complicated crown fractures in young permanent teeth, its efficacy in mature permanent teeth requires further validation [9]. Research has shown that the success rate of pulp capping decreases significantly in teeth with crown fractures and lateral displacement, as the periapical blood supply is compromised [13, 14]. This study excluded teeth with displacement injuries such as partial extrusion, lateral displacement, and intrusion.

This prospective cohort study evaluates the long-term outcomes of pulpotomy using iRoot BP Plus for nondisplaced complicated crown fractures in permanent anterior teeth of children.

## 2. Materials and Methods

This study has been written according to Preferred Reporting Items for Observational Studies in Endodontics (PROBE) 2023 guidelines [15]. This study was approved by the Ethics Committee of Peking University School and Hospital of Stomatology (PKUSSIRB-202169169) and registered in the Chinese Medical Research Registration System with Number MR-11-23-050481 (www.medicalresearch.org.cn). Teeth diagnosed with complicated crown fractures that were treated from November 2020 to December 2024 were collected in this study. All teeth were treated according to the clinical routine for pulpotomy.

Inclusion criteria are as follows:

1. Children without systemic diseases who could cooperate with the treatment, with informed consent signed by their parents.
2. Crown fractures with pulp exposure, without root fracture and displacement, no percussion pain or slight percussion pain (+ or ++).
3. X-ray showing continuous and intact periodontal ligament, possible widening of the periodontal ligament, no periapical low-density images, and no internal or external root resorption.
4. At least one follow-up record.

Exclusion criteria are as follows:

1. Patients with systemic diseases, allergies to local anesthetics, or the pulp capping agent used.
2. Teeth previously treated in other hospitals.
3. Teeth with displacement injuries (extrusive luxation, lateral luxation, and intrusive luxation).
4. Inability to achieve hemostasis with a 3% sodium hypochlorite cotton ball under light pressure for 3 min after infected pulp removal.

At the initial visit (*T*), each patient underwent a visual examination, probing, percussion, mobility assessment, a cold pulp vitality test, and periapical radiography. The decision to perform a partial pulpotomy or full pulpotomy was based on the fracture area, the exposed pulp site, and the bleeding condition of the tooth's pulp during the procedure.

### 2.1. Sample Size

The sample size was calculated based on the results of Hu, who reported a 97.7% success rate for iRoot BP Plus pulpotomy in permanent incisors with complicated crown fractures [16]. Based on the effect size method, with an *α* of 5% and a power of 80%, a minimum of 30 participants was required to ensure statistically significant results. To account for an expected attrition rate of approximately 20%, 36 patients were initially planned for inclusion in the study.

### 2.2. Pulpotomy Procedure

Six senior dentists performed all pulpotomy procedures after receiving standardized protocol training [17]. Under local anesthesia with 4% articaine with epinephrine (1:100,000), a rubber dam was placed. Two millimeters of pulp around the exposed area were removed by a high-speed diamond round bur, followed by rinsing with 3% sodium hypochlorite solution. Hemostasis was then achieved by lightly pressing with a sterile wet cotton ball for 3 min. If hemostasis could not be achieved, additional pulp was resected until all coronal pulp was removed and hemostasis was achieved. Additionally, teeth with crown fractures involving more than two-thirds of the crown underwent full pulpotomy from the outset. A minimum 2 mm thick layer of iRoot BP Plus (Innovative BioCeramix Inc., Vancouver, BC, Canada) was then applied to cover the pulp stumps, followed by a base of Fuji IX glass ionomer (GC Corporation, Tokyo, Japan). If the tooth fragment is available and intact, reattachment was performed. Otherwise, the tooth was restored with a resin filling according to the defect area.

### 2.3. Follow-Up

The follow-up was scheduled according to the time recommended for radiographic examination in the clinical guidelines for dental trauma, at 6–8 weeks (T1), 3 months (T2), 6 months (T3), and 1 year (T4) after the injury, with routine check-ups thereafter [18]. The follow-up period was calculated based on the last outpatient visit.

Clinical examination included crown color; probing; percussion; mobility; cold pulp vitality test (not applicable for patients after full coronal pulp removal, as there was no vital coronal pulp); and periodontal condition.

Radiographic examination assessed the continuity of the periodontal ligament, periapical low-density areas, formation of a dentin bridge beneath the capping material, and calcification of the pulp chamber and root canal.

Clinical and radiographic evaluation criteria for pulpotomy are shown in Table 1. Treatment success was defined by the presence of both clinical and radiographic success criteria. Failure was defined as any case not meeting all specified success criteria. Clinical or radiographic failure was considered an endpoint event, and the time of its occurrence was recorded as the endpoint time.

### 2.4. Statistical Analysis

Analysis was conducted using IBM SPSS 22.0 statistical software. Categorical variables were analyzed using frequencies and percentages, while continuous variables were described using means and standard deviations for descriptive statistics. Kaplan–Meier survival analysis was used to estimate the chance of survival over time. The chi-square test or Fisher's exact test was used to evaluate the influence of factors affecting the success rate. *p*  < 0.05 was considered statistically significant.

## 3. Results

A total of 126 permanent incisors in 107 patients were included in the study. Among them, 80 teeth were from 71 males and 46 teeth were from 36 females. The average age of participants was 10.4 ± 2.0 years, and the mean follow-up time was 14.1 ± 12.6 months. Among the incisors, 67 were young, immature permanent teeth, while 59 exhibited mature root development. Full pulpotomy was performed on 86 teeth, whereas 40 underwent partial pulpotomy. Treatment timing post-trauma was distributed as follows: ≤12 h (*n* = 73), 12–24 h (*n* = 30), and >24 h (*n* = 23). Baseline characteristics and tooth distribution are detailed in Tables 2 and 3, respectively. The most commonly affected teeth were 11 and 21, comprising 42.1% (53/126) and 52.4% (66/126) of cases. Twenty-five cases had concomitant injuries with subluxation (Table 2).

**Figure 1:**
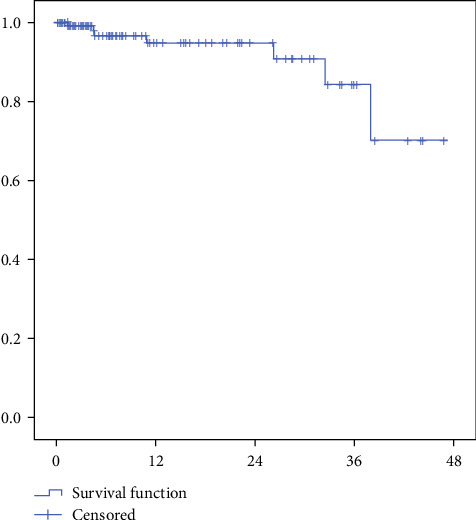
Survival estimates of traumatic incisors postpulpotomy over time. The “vertical line across the curve” denotes “Censored” data, which refers to subjects who did not experience the failure event during the study period.

**Figure 2:**
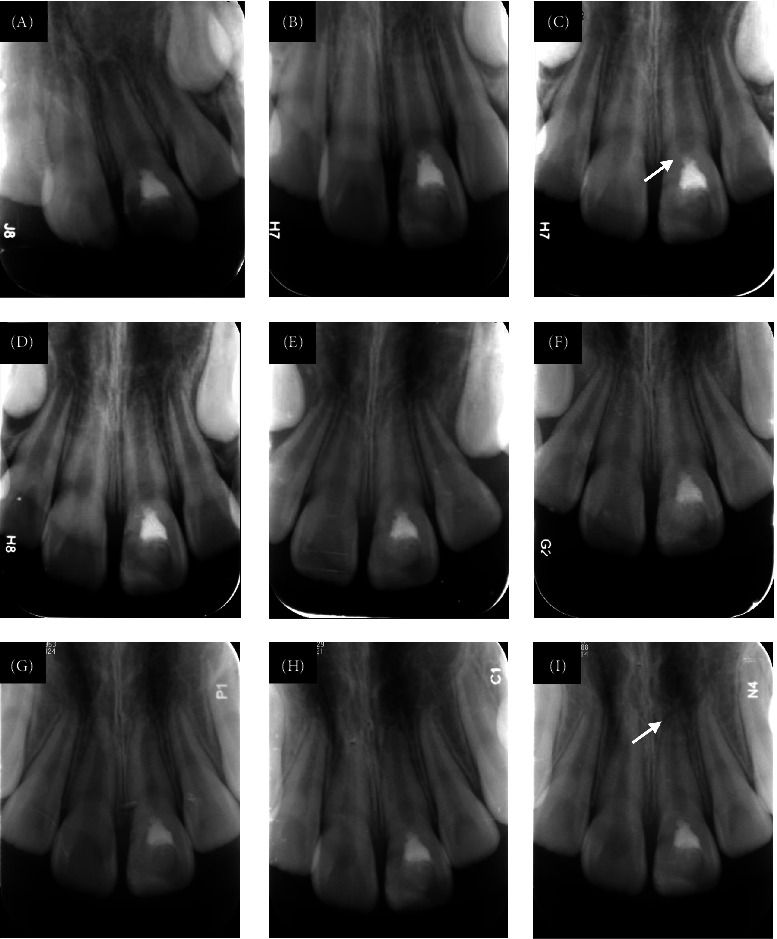
Periapical radiographs of tooth 21 of a 7-year-old girl with a complicated crown fracture and immature root formation that underwent full pulpotomy. (A) Immediate postoperative radiograph. (B) Follow-up radiograph at 1 month. (C) Follow-up radiograph at 3 months, white arrow points to the dentin bridge. (D–H) Follow-up radiographs at 6, 12, 18, 24, and 30 months. (I) Follow-up radiograph at 44 months showed that the apical foramen was closed (white arrow).

**Figure 3:**
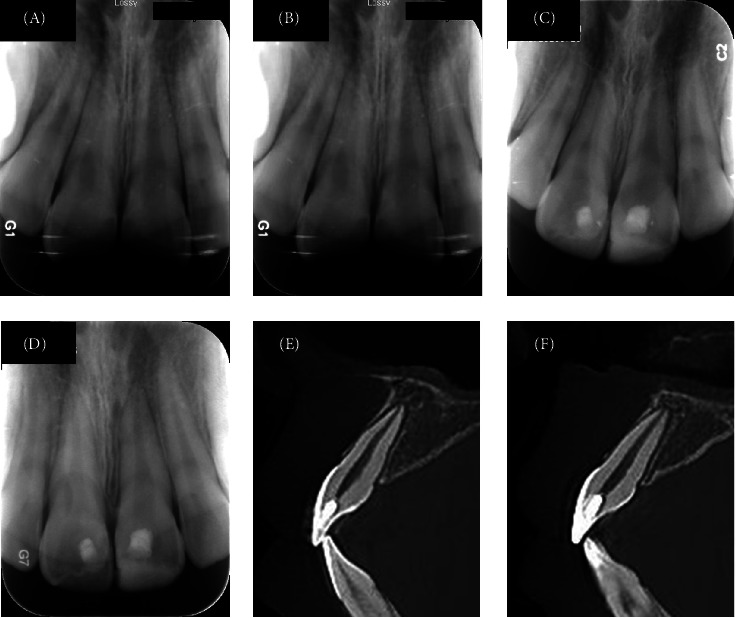
Upper central incisors of a 12-year-old boy with complicated crown fractures and immature root formation that underwent partial pulpotomy. (A) The preoperative periapical radiograph. (B–D) The 3-, 15-, and 24-month follow-up periapical radiographs. (E–F) The sagittal plane of CBCT images at 24-month follow-up.

As of May 31, 2025, success rates stood at 95.9% (70/73) at 6 months and 92.3% (48/52) at 12 months (Table 4). Kaplan–Meier survival analysis estimated a 94.7% ± 2.7% survival rate at 2 years post-treatment (Figure 1). This indicates that >90% of treated teeth maintained pulp vitality and function without requiring root canal treatment during this period - a critical timeframe where most complications typically emerge. In the radiographic evaluations, no signs of periradicular bone or root resorption were observed in successfully treated teeth. A hard tissue bridge underneath the iRoot BP Plus layer was observed on radiographic examination in all successful cases with follow-ups exceeding 6 months. Crown discoloration was not noted in any of the reviewed cases. Typical successful cases are illustrated in Figures 2 and 3.

**Figure 4:**
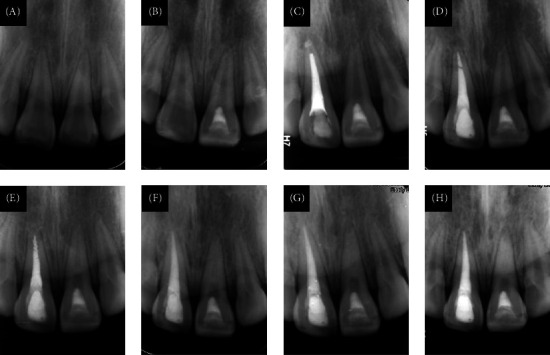
Tooth 21 of a 13-year-old boy diagnosed as a complicated crown fracture combined with subluxation and mature root formation that underwent full pulpotomy. (A) The preoperative periapical radiograph. (B–C) The 3- and 4-month follow-up periapical radiographs. (D) The radiograph at the 7-month follow-up showed external root resorption and a suspicious periapical lesion of tooth 21. (E–H) The radiographs at the 20-, 26-, 32-, and 38-month follow-ups. Spontaneous healing of the periapical lesion was observed, despite worsening pulp canal calcification.

Notably, one case involving tooth 21 with subluxation developed external root resorption and a periapical lesion 7 months post-treatment (Figure 4A–D). However, spontaneous healing was observed at the 20-month follow-up, despite worsening pulp canal calcification, culminating in pulp canal obliteration by 38 months (Figure 4E–H).

In this study, there were a total of seven failures, resulting in an overall success rate of 94.4% (119/126) (Table 5). Failures were attributed to periapical periodontitis. Although eight cases experienced filling loss requiring repair, none of them were associated with further pulp inflammation. Statistical analysis indicated that factors such as gender, root maturity, pulpotomy type, pulp exposure time, and tooth mobility did not significantly affect success rates (*p*  > 0.05) (Table 2).

## 4. Discussion

Our findings demonstrate high success rates (95.9% at 6 months, 92.3% at 12 months, estimated 94.7% ± 2.7% at 2 years) for pulpotomy using iRoot BP Plus in nondisplaced complicated crown fractures of permanent anterior teeth in children. This aligns with recent studies reporting success rates of 75%–96% for bioactive ceramics in similar trauma [1, 16, 19], and specifically mirrors the 90.0% success rate for iRoot BP Plus partial pulpotomy in immature teeth reported by Yang et al [19]. Notably, our results extend this high efficacy to mature permanent teeth (59 cases), with success rates comparable to immature teeth (*p*  > 0.05, Table 2), supporting the concept that root maturity does not preclude vital pulp therapy in carefully selected traumatic cases [1, 20].

Our study demonstrates that pulpotomy using iRoot BP Plus achieves excellent long-term outcomes for pediatric complicated crown fractures, with an estimated 94.7% 2-year survival rate. This indicates most treated teeth maintain vitality without progressing to root canal treatment, particularly valuable for growing patients where preserving natural tooth development is crucial. The stability of results (95.9% at 6 months vs. 92.3% at 12 months) suggests failures typically manifest early, underscoring the need for close initial follow-up. Compared to alternatives, iRoot BP Plus demonstrates superior success rates to calcium hydroxide while matching MTA and Biodentine's efficacy [16, 20–22]. Its clinical advantages are noteworthy: easier handling compared to MTA's gritty consistency and no risk of tooth discoloration, a significant esthetic consideration for anterior teeth [16, 21]. These biological and practical benefits establish iRoot BP Plus as an optimal choice for pediatric dental trauma, though multicenter comparative studies could further validate these observations.

In this study, most patients (89 cases) visited the clinic within 24 h after trauma. When the crown is severely fractured, exposing the pulp to the contaminated oral environment, early consultation is crucial for infection control. Interestingly, neither the time elapsed between trauma and treatment (≤12, 12–24, >24 h) nor the extent of pulp removal significantly influenced success rates in our cohort (*p*  > 0.05, Table 2), which is confirmed by many studies [1, 16]. Complex crown fractures should be treated as emergencies, but pulpotomy can also be performed after 24 h [23]. In this study, 23 cases were treated more than 24 h after trauma, with the longest delay being 12 days, yet clinical and radiographic success was still achieved. While dental trauma is an emergency, teeth not treated immediately may still preserve living pulp, suggesting the pulp's inherent ability to resist infection.

The exclusion of teeth with displacement injuries (extrusive, lateral, intrusive luxation) aligns with established evidence linking displacement to increased pulp necrosis risk due to compromised apical vasculature [13, 14, 24]. While concomitant subluxation injuries (indicating periodontal involvement) were present in 25 cases, they did not appear to significantly affect success rates in our analysis (*p*  > 0.05, Table 2). However, the small subluxation subgroup limits definitive conclusions on its independent effect. It has been reported that crown fracture increases the risk of pulp necrosis in teeth with concussion or subluxation injuries [25, 26]. Root canal obliteration (pulp canal obliteration [PCO]) is a common complication of dental trauma and pulpotomy [1, 27]. One case (tooth 21 with subluxation and mature root) developed external root resorption and a periapical lesion at 7 months (Figure 4A–D) but showed spontaneous periapical healing by 20 months despite progressive PCO (Figure 4E–H). Consistent with our definition (success = absence of apical pathology, no need for pulpectomy), this case was considered successful at final follow-up, reflecting the ongoing debate on classifying PCO as an outcome [1, 28]. Future studies with larger samples and longer follow-up periods are needed to understand the complex relationships between different types of tooth injuries.

Eight cases experienced filling loss requiring repair. Importantly, radiographic evidence of dentin bridge formation in successful cases suggests coronal filling loss did not provoke pulp inflammation. Because resin repair is considered a temporary solution, minimal preparation was performed to preserve more of the tooth's natural tissue, and resin filling was done according to the beveled surface of the defect. While this method reduces the risk of the filling falling out, it is esthetically suboptimal. However, children have esthetic needs for their anterior teeth and maintaining social interactions, highlighting the necessity for further exploration of new transitional repair methods. In three cases, crown reattachment was performed. If the broken crown is found and preserved correctly, reattachment can serve as a temporary or even permanent restoration for anterior tooth trauma [29]. Research has indicated that the bonding modes “internal concavity + lingual relief path + buccal hole slope” and “internal concavity + buccal slope” are the most effective for bonding anterior tooth crown fractures. With appropriate tooth preparation and a suitable bonding system, crown reattachment can achieve a long-term clinical retention rate [30].

Long-term follow-up of traumatic teeth is crucial. However, more than half of the patients, for various reasons, were unable to adhere to regular check-ups as per clinical guidelines. Despite this, due to the irreversible nature of endpoint events, patients who missed interim check-ups but rejoined follow-ups at T4 or later and showed successful outcomes in both clinical and radiological examinations demonstrate that the treatment remained successful during the unmonitored period.

All 7 failed cases in this study developed periapical periodontitis requiring pulpectomy to halt inflammatory spread and preserve buccal bone integrity. Notably, 3 of these failures were asymptomatic at earlier follow-ups, reinforcing that chronic periapical pathology may lack overt symptoms [31]. This underscores a critical finding from our cohort: regular radiographic monitoring is essential even in asymptomatic teeth, especially given that >50% of patients missed scheduled visits. Future implementation of electronic medical record alerts could mitigate this by automating recall reminders.

This study has several limitations to consider. As a single-arm prospective cohort, it lacks a control group for direct comparison (e.g., MTA). Variable follow-up durations (range: 1–44 months) create unequal observation periods, where teeth lost to follow-up might have undetected late failures. Although we used Kaplan–Meier analysis to account for censored data, the wide standard deviation (±12.6 months) indicates inconsistent monitoring intervals that could mask time-dependent failure patterns. The observed decline in survival rates beyond 3 years (Figure 1) suggests the need for extended follow-up studies to evaluate long-term outcomes more comprehensively. Although procedures were standardized and performed by experienced clinicians within one institution, the inherent variability in operator technique and patient compliance could influence results. While the exclusion of displaced teeth intentionally controlled for vascular compromise risk, this selection criterion, combined with the limited sample size of concomitant subluxation cases (*n* = 25), may introduce selection bias and consequently limit the generalizability of our findings to broader trauma populations, potentially overestimating success rates for more severe injury patterns. Future randomized controlled trials with extended follow-up, multicenter designs, and inclusion of diverse trauma patterns are needed to confirm and expand upon these results.

Collectively, these findings demonstrate that iRoot BP Plus pulpotomy achieves predictable outcomes for nondisplaced complicated crown fractures in pediatric permanent teeth, independent of root maturity or treatment timing. The high success rates (exceeding 90% at 1 year) support its adoption as a primary treatment modality. Future studies should address transitional restorative challenges, sample size, and validate long-term outcomes beyond 3 years, particularly in teeth with concomitant injuries.

## 5. Conclusions

Pulpotomy using iRoot BP Plus is a highly successful conservative treatment for nondisplaced complicated crown fractures in pediatric permanent anterior teeth. Prognosis remains favorable regardless of root maturity, pulpotomy technique (partial/full), or time-to-treatment post-trauma.

## Figures and Tables

**Table 1:** Clinical and radiographic success evaluation criteria.

Success criteria	Clinical success criteria	Radiographic success criteria
1	The tooth had no subjective symptoms	No pathological root resorption on periapical radiographs
2	The crown color was normal	No periapical low-density
3	Percussion test (−) or (±), no pathological mobility	Continued development of the root in young permanent teeth
4	No gingival swelling or sinus tract	Formation of a dentin bridge

*Note:* Calcification of the pulp chamber and root canal was not considered a failure.

**Table 2:** The baseline characteristics of the study participants.

Variables	Category	Number	Success rate (%)	*p*-Value
Gender	Male	71	—	—
	Female	36	—	—

Root maturity	Mature	59	98.3	—
	Immature	67	91.0	0.207

Pulpotomy	Full pulpotomy	86	91.9	—
	Partial pulpotomy	40	100	0.096

Pulp exposure time	≤12 h	73	93.2	—
	12 < *T* ≤ 24 h	30	96.7	—
	>24 h	23	95.7	0.728

Tooth mobility	0	101	95.0	—
	1	21	95.2	—
	2	4	75.0	0.272

**Table 3:** The distribution of tooth position.

Position	11	21	31	41	22	32
Number	53	66	3	1	1	2
Percentage (%)	42.1	52.4	2.4	0.08	0.08	1.6

**Table 4:** The success rate of follow-up at each time after trauma.

Follow-up time	T1	T2	T3	T4
Success rate (%)	99.2	99.0	95.9	92.3
Tooth number	125/126	97/98	70/73	48/52

*Note:* 6–8 weeks after trauma (T1), 3 months after trauma (T2), 6 months after trauma (T3), 1 year after trauma (T4).

**Table 5:** Seven failed patients' demographics in the study.

Patient no.	Age (years)	Gender	Teeth number	Tooth mobility	Pulpotomy type	Apex	Fail time (months)
1	8.9	Male	11	0	Full	Open	4.5
2	12.1	Male	11	1	Full	Close	26.2
3	8.9	Male	21	0	Full	Open	1.4
4	9.2	Female	21	0	Full	Open	10.9
5	8.3	Male	11	0	Full	Open	32.5
6	7.8	Female	21	2	Full	Open	4.6
7	8.0	Female	11	0	Full	Open	38.0

## Data Availability

The data that support the findings of this study are available upon request from the corresponding author. The data are not publicly available due to privacy or ethical restrictions.
